# Reducing health inequities affecting immigrant women: a qualitative study of their available assets

**DOI:** 10.1186/s12992-016-0174-8

**Published:** 2016-07-07

**Authors:** Anna Bonmatí-Tomás, Maria del Carmen Malagón-Aguilera, Cristina Bosch-Farré, Sandra Gelabert-Vilella, Dolors Juvinyà-Canal, Maria del Mar Garcia Gil

**Affiliations:** Nursing Department, Faculty of Nursing, University of Girona, Emili Grahit, 77, 17003 Girona, Catalonia Spain; Health and Health Care Research Group, University of Girona, Girona, Catalonia Spain; Director of Health Promotion Chair, University of Girona, Pic de la Peguera 15. Parc Científic i Tecnològic, 17003 Girona, Catalonia Spain; Research Unit of Family Medicine Girona (Vascular Health Group), Primary Care Research Institute Jordi Gol, Girona, Spain; TransLab Research Group, Department of Medical Sciences, School of Medicine, University of Girona, Maluquer Salvador, núm. 11, 17002 Girona, Catalonia Spain

**Keywords:** Salutogenesis, Assets, Immigrant women, Health inequities, Salutogénesis, Activos en salud, Mujeres inmigrantes, Inequidades en salud

## Abstract

**Background:**

Immigrant women often experience health inequities, whether for reasons of gender, country of origin, or socioeconomic status. The view of immigrant women has always focussed on their needs, without taking into account their available assets. A salutogenic approach incorporating an assets analysis could provide a new perspective on the design of health promotion interventions to reduce health inequities. The study objective was to identify the assets of this group of women as a necessary first step in changing the paradigm used in such health promotion interventions.

**Methods:**

This qualitative study combined focus groups, in-depth interviews, and a photovoice session. The aim was to describe the assets of this group, based on Antonovsky’s salutogenic approach and assets model. Qualitative results were interpreted with a phenomenological focus, identifying each individual’s internal, community, and institutional assets.

**Results:**

The self awareness of skills was linked to a person’s description of herself as being optimistic, having religious beliefs, and having motivations and objectives in life, for herself, her family or her children. Being motivated helped the women to persist in doing or learning things that could be useful in confronting difficult situations. Another selfawareness skill was feeling useful to others, whether this was due to religious beliefs about their role in life or to the importance of the mutual support of interpersonal relationships.

**Conclusions:**

High optimism, strong capacity for struggle and self-initiative, the importance of religious beliefs, social support, and concern for their children’s future were described as assets of immigrant women.

Identification of these assets allows us to develop more in-depth knowledge and better tools for health promotion programs and policies intended to reduce health inequities in this population of immigrant women.

## Resumen

**Antecedentes:**

Las mujeres inmigrantes a menudo experimentan inequidades en salud por razón de género, país de origen o estado socioeconómico. La visión de las mujeres inmigrantes siempre se ha focalizado desde sus necesidades no teniendo en consideración los acticos de éstas. La salutogénesis y el análisis de los activos en salud pueden dar un nuevo enfoque en el diseño de las intervenciones de promoción de la salud para reducir las inequidades en salud. El objetivo de este estudio fue identificar los activos de este colectivo de mujeres como primer paso para poder reorientar las intervenciones de promoción de la salud.

**Metodología:**

Este estudio cualitativo combina sesiones de grupos focales, entrevistas en profundidad y una sesión de fotovisión. El objetivo fue describir los activos de este grupo basándonos en la aproximación salutogénica de Antonosvky y el modelo de activos en salud. Los resultados cualitativos fueron interpretados con un enfoque fenomenólógico, identificando los activos internos individuales, comunitarios y los institucionales.

**Resultados:**

El autoreconocimiento de habilidades junto a la autodescripción de las participantes como personas con un elevado optimismo, creencias religiosas y con motivaciones y objetivos en la vida para ellas, su familia o sus hijos. Esta motivación ayuda a las mujeres a esforzarse en realizar o aprender aquello que pueda ser de utilidad para hacer frente a situaciones difíciles. Otra habilidad autoreconocida es el sentirse útil a los demás, bien sea por sus creencias religiosas o por la importancia del apoyo mutuo de las relaciones interpersonales.

**Conclusiones:**

Los activos descritos por las mujeres inmigrantes fueron un elevado optimismo, la gran capacidad de lucha y iniciativa propia, la importancia de las creencias religiosas, el soporte social y la preocupación por el futuro de sus hijos.

La identificación de estos activos nos permite profundizar el conocimiento y desarrollar mejores instrumentos para las políticas y los programas de promoción de la salud cuyo objetivo es la reducción de las inequidades de salud en la población de mujeres inmigrantes.

## Background

### Health inequities affecting immigrant women

The current economic crisis in Europe has notably increased inequality between social classes [1]. A key concept of the World Health Organization’s Commission on Social Determinants of Health is that avoidable inequalities result in health inequities between different populations [2]. The most vulnerable groups, including immigrants, have been most affected, directly and/or indirectly [3]. The majority of the immigrant population has low socioeconomic status, given their lack of economic resources, and immigrants are among the groups most vulnerable to social exclusion.

Eurostat Data defines immigrant populations as “people arriving or returning from abroad to take up residence in a country for a certain period, having previously been resident elsewhere” [4]. On the other hand, some authors believe that this definition should specify voluntary movements, without external pressures [5] i.e., involuntary migrations of refugees due to wars or natural disasters. In Europe, the immigrant populations have a fundamental role in modifying the population pyramid as the native population ages, in addition to making rich multicultural contributions [6].

Europe received nearly 2 million immigrants in 2013, for a total immigrant population of about 34 million in 2014 [4]. Immigrants constitute 7.8 % of the total population of Spain, 14.5 % in the autonomous community of Catalonia, and 18.7 % in the study area, Girona Province [7, 8].

Immigrant populations are diverse, both in terms of the reasons for their arrival in a new country and their experiences as immigrants [9]; among the immigrant populations arriving in Catalonia over the past decade, using the Eurostat definition, the top three countries of origin are Morocco (25 %), Honduras (14.6 %), and Gambia (8 %) [8]. The average age of immigrants in Catalonia is 32 years; however, this working-age population has a high rate of unemployment: 34.2 %, compared to 17.6 % of the native population [10]. Therefore, the majority of the immigrant population faces precarious employment, unemployment, fewer employment opportunities and discrimination in the hiring process [11], a situation that is confined to the lowest socioeconomic levels. There is a known relationship between low socioeconomic status and increased risk factors for poor health such as smoking, obesity, and low perceived quality of life [12]. A relationship has also been observed between unemployment and harmful effects on health, including increased mental diseases and unhealthy lifestyle habits [13, 14].

The social integration of immigrants is complicated by major cultural differences [15]. This has been called acculturative stress, defined as a decline in mental health and wellbeing that occurs during the process of adapting to a new culture [16]. Among the stressors that have been described in the immigrant population, the key factors are linguistic and communication barriers, sociocultural changes, economic concerns, unemployment, and marginalization [17, 18], in addition to the loss of family and social support available in the country of origin. Moreover, the immigrant population often experiences social exclusion, defined as negative attitudes, rejection, or ostracism by others without an explicit reason. This social exclusion generates strong feelings of rejection, anxiety, and social problems, and can affect the four basic needs of belonging, self-esteem, control, and meaningful existence [19].

Immigrant women are specifically at high risk of gender-related health inequities [20], including worse health status [14] and increased morbidity and mortality [21], compared to men. Additionally, high levels of stress and low self-esteem have been reported as characterizing immigrant women [22]. Self-esteem reflects the general opinion each person has of his or her positive and negative attributes and is also considered a protective factor that contributes to better health and social wellbeing in confronting negative situations [23]. Social support, which facilitates integration and adaptation to the new culture and surroundings, can be another protective factor for immigrant women [24].

Given the daily realities facing immigrant women, the present study attempted to offer a new perspective, focussing on the positive assets of this population group and the ways these assets can help them to cope with the stressful situations and health inequities they face.

### Salutogenic approach and assets model

The salutogenic approach developed by Aaron Antonovsky offers this positive perspective and emphasizes those things that create health, in contrast to the pathogenic approach, which focuses on the problems and obstacles faced in achieving health [25]. Health is considered a dynamic configuration of social, personal and physical resources [26], a continuous “ease/disease” process [27]. An individual can be considered as being between two poles on a continuum from health to disease or, in Antonovsky’s play on words, from ease to disease [28]. Later authors have referred to these two endpoints as total health and the absence of health [29]. Strümpfer criticized the salutogenic model, alleging that placing “health” at the end of the continuum is a limitation of the theory and suggesting that a broader construct is needed, such as quality of life [30].

Antonovsky defined two constructs: Sense of Coherence (SOC) and General Resistance Resources (GRR). The first is “a global orientation that expresses the extent to which one has a pervasive, enduring though dynamic feeling of confidence that (1) the stimuli from one’s internal and external environments in the course of living are structured, predictable, and explicable; (2) the resources are available to one to meet the demands posed by these stimuli; and (3) these demands are challenges, worthy of investment and engagement” [25]. Some authors have argued that there are similarities and convergences between the concept of SOC and several other theories, including Rotter’s locus of control [31], self-efficacy as described by Bandura [32] or learned resourcefulness developed by Rosenbaum [33].

The second construct, GRR, encompasses the characteristics of a person, group, subculture, and/or society that can effectively combat a wide variety of stressors. These resources may be inherent to a person or his/her abilities, to the immediate or distant surroundings as a material or psychological quality of the person, or to the larger society, such as money, housing, self-esteem, knowledge, heritage, and attitudes about health, social relationships, existential questions, beliefs, religion, and meaning of life [25, 27]. Not only is it important to have these resources, but also for the individual to be able to identify and be able to use them. The stronger a person’s SOC, the greater his or her ability to identify and use these resources [34].

More recently, Morgan and Ziglio defined an assets model, based on the salutogenic approach and other methods [35]. An asset has been defined as any factor or resource that strengthens the capacity of individuals and communities to maintain health and well-being, attempting to emphasize positive skills and the capacity to identify problems and act on solutions in order to promote individual self-esteem and community leadership in improving health [36]. At the individual level, the authors list as assets: self-esteem, positive values, social skills, and resiliency. Community-level assets include family support networks, community cohesiveness, tolerance among community members, and volunteer association. Finally, organizational or institutional assets include social justice, democracy, and the material resources needed for physical activity. In contrast to the pathogenic approach, focused on deficits, this assets model identifies factors that promote health and wellbeing, make the population an active participant in creating its own health status, empowers individuals to engage in self-care, and contributes to more sustainable social and economic development [35].

Some authors consider assets to have many similarities to other concepts, such as resiliency and resourcefulness. Resilency, for example, shares some but not all of the characteristics of the assets model. On the other hand, resourcefulness is more related to the consequences of health assets than to the assets themselves [37]. Other authors have discussed the similarity between GRR and assets, concepts drawn from different theoretical models that encompass the same construct [38]. In the present study, GRR and assets were explored as a combined concept.

A few recent studies in populations of women experiencing inequality have focused on assets, not only on deficits and stressors. Some of these authors have described and assigned value to the role that these women can fulfill in their communities [39, 40]. The objective of the present study was to describe the GRR or assets of immigrant women, providing a positive view based on a salutogenic approach. This new, positive approach reveals their assets as a highly valuable tool in refocusing interventions intended to reduce the health inequalities faced by this population group.

## Methods

### Study design

We designed a qualitative method study with a phenomenological focus and a variety of data-gathering techniques [41]. The phenomenological focus allows a description of experiences and an awareness of the facts, without looking for causes [42]. The study was carried out in Girona (Spain), from March to July 2014 in a work preparedness program for women, a project of the Girona office of Caritas, the international Catholic Charities organization that works with unemployed and homeless individuals and families and with immigrants.

### Recruitment

One of the researchers explained the project to participants in the Caritas program, in clear, uncomplicated terms, and responded to all of the questions the women wanted to ask, as a group and individually as needed. Those who agreed to voluntary participation in the project understood that the only potential benefit was the opportunity to better understand themselves [43]. A convenience sample of 8 immigrant women aged 25 to 50 years was recruited to participate in the study. According to some authors, qualitative studies can reach data saturation with 7 or 8 participants [44].

### Participants

The participants were residents of Catalonia from 6 different countries of origin in three regions of the world. Three were from sub-Saharan Africa: the youngest of all the participants (JF) is Senegalese, without a stable partner and unemployed for 1 year; another (SM) is from Burkina Faso, has 2 daughters, her husband is employed half-time, and she works a few hours a week cleaning a house; the third (HS) is from Ghana and has lived in a city shelter for 2 months because she has no source of income. Three were from Central America: the oldest of the whole group (CM) is unemployed and all her close family members remain in her home country, Ecuador; another from Ecuador (LA) has a husband and son in Girona and although her socioeconomic level is higher than the rest of the group, she has had problems with social integration; a married woman from Honduras (XB), with three children younger than 10 years, is active in the protestant Evangelical Church in Girona. Two of the participants were from Morocco: one (KS) is divorced and lives with her daughter in an apartment offered by Caritas; the other (BD) lives with her husband, who has been unemployed for almost a year, and two children in their own house.

### Data collection

The research was carried out in a community room at the town hall, in order to dissociate the study from Caritas and communicate to participants that they could express their opinions and feelings without fear of judgment, with total trust, and with confidence that their participation would not affect their relationship with the work preparedness program. All participants gave signed informed consent and agreed to maintain the confidentiality of everything that was said during the focus group sessions. The study was carried out in accordance with the Declaration of Helsinki and Council for International Organizations of Medical Sciences CIOMS guidelines for research in vulnerable populations [45]. The Committee on Clinical Research Ethics of Dr Josep Trueta University Hospital in Girona (Spain) approved the study (2014/029).

Data were collected using conceptual, metaphoric language, and information was captured in a flexible and unstructured fashion, using procedures that are more inductive than deductive and a holistic orientation that leads to a broader and deeper awareness of the topics of interest [46]. Given the difficulty of identifying health assets for each individual, we also chose to apply several different qualitative techniques –a focus group, photovoice session, and in-depth interview– in order to encourage self-awareness, surface information, and collect the data of interest [41]. In order to establish greater trust and empathy with the participants, all of these techniques were carried out by the same researcher. All participants understood that no information they provided would be shared with Caritas, their comments would be identified by randomly generated initials (not their own), and the study results would not be published until they had completed the work preparedness program.

First, we carried out 2 focus group sessions with all participants, lasting from 1.5 to 2 h. Two sessions were required because of some participants’ limited proficiency in Spanish, which required more time to allow them to express themselves to the group. These open-ended sessions dealt with various themes: life goals and plans, family and social relationships, their own roles and contributions, and other topics that arose spontaneously and generated interest among the participants. For some, a focus group enriches the discourse in multicultural settings [47, 48], and it is also valued as a co-learning tool for participants that facilitates clarification and understanding of the phenomenon being studied [49].

In addition, an adapted photovoice session was held. This technique is defined as “a process by which people can identify, represent, and enhance their community through a specific photographic technique” [50]. It has three goals: it enables the use of photographs to show the needs and assets of a community, which increases individual empowerment regardless of the participant’s language skills [51], it promotes critical thinking, and it offers participants a means to reach policymakers. Some authors have described it as a process that allows people to put words to images, helping the most vulnerable to narrate their own stories and describe the realities of their daily lives [52].

In the context of the present study, photovoice allowed discussion of participants’ views and perceptions of topics such as their role in the family and community and their goals and plans for the future. The photovoice session began with the participants sharing three photographs taken on their cell phones to show what was most important in their life. Next, they discussed their choices with the group, explaining the importance of each photo to them and the dreams reflected in each one.

Finally, in-depth interviews were carried out with each participant. These open-ended conversations allowed each woman to comment further, and more privately, on topics that surfaced during the focus groups and photovoice session, or to raise new topics of her own. During each interview, the researcher attempted to achieve a high level of empathy with each individual, control the emotional tone at all times, and ask the most sensitive or difficult clarifying questions at the opportune time [53].

The researcher kept a field diary during the study, where memos on all of the steps taken were kept, along with personal observations that might be considered relevant. The collected data were not included in the results because they were considered relevant only during the process itself.

### Data analysis

The focus group sessions, adapted photovoice session, and in-depth interviews were all self-recorded and transcribed verbatim. Data from the focus groups and photovoice session were analysed in a first phase, and personal interviews were carried out and analysed in depth in a second phase, until data saturation was achieved.

All sessions were categorized and analysed using Atlas Ti v.7 software. Data analysis was carried out by 3 researchers, who independently integrated all qualitative techniques in 4 phases: general analysis, definition of codes, line-by-line coding, and disaggregation of certain codes (Table 1). Consensus was reached on all coding decisions. A second analysis of the results was carried out to identify relationships between the assets identified.

**Table 1:** Phases of qualitative analysis

Phase	Activity
Phase 1:	General analysis of each interview to establish sociodemographic data for each participant and preliminary general coding of the most notable characteristics of the interview
Phase 2:	Definition of the codes applied, based on previous knowledge and the study objectives
Phase 3:	Line-by-line coding of each interview
Phase 4:	Review of Phase 3 coding to disaggregate codes as appropriate.

## Results

Results obtained from the integration of the diverse qualitative methodologies were grouped by considering the person at the centre of the study and taking into account the various determinants of health. We followed the assets classification system proposed by Morgan and Ziglio, identifying each one as an individual, community or institutional asset.

### Individual assets

#### Ability to manage problematic situations

These women are not afraid to confront complex or challenging situations. It forms part of their daily life.

*“I talk to the king like I talk to the president. I don’t bite my tongue for anything; if a person can help me, I just go along. I am a person that does what I say I will do.” XB*

*“But I am very strong in my heart. I am a fighter.” HS*

*“I have always been a woman who fights to move everything forward, because I’ve had bad luck; I had a husband who didn’t worry about much and I always had to move everything forward.” BD*

#### High optimism

Positive and hopeful thoughts about life help them to survive the situation of vulnerability in which they find themselves:

*“I have always been a positive person and I have met all the challenges I wanted to. I have met them.” XB*

*“This is an opportunity; I look at it that way. It is an opportunity, a gift from God and we’ll see what happens.” LA*

*“No, I don’t think negatively, I think positive and I think I will find work that is what I have in my head.” SM*

#### Self-awareness of skills

Having appreciation of their own manual, social and other useful skills, such as cooking or sewing, and/or of their highly developed and important role in the family is a positive asset.

*“I love to bake bread, and on Saturday and Sunday afternoons, I love to make things, like bread for tea-time.” KS*

*“I’m divorced now, so now I do everything, I’m father and mother.” KS*

#### Initiative

These women recognize that they have taken the initiative in making vital decisions in their own lives or took the lead in managing situations that were difficult for them:

*“We didn’t like living in Ecuador, we were overwhelmed. But my husband didn’t do anything about it, he wasn’t sure about anything. One day I just said, “Come on, let’s go to Europe because I have a cousin there who tells me that she has found a good life.” LA*

*“Yes, taking a step from one country to another, you have a plan and clear ideas.” CM*

#### Religious beliefs

Faith is important to being able to remain optimistic and be hopeful, in order to be able to manage tensions and stress or not feel alone when facing difficulties:

*“I am very much a believer, God first; first God goes with me, if he does not go with me, I don’t go, I stay.” CM*

*“Yes, because not everybody has work, but with patience, cheerfulness, with … faith. I have faith.” HS*

#### Motivations, life goals and/or objectives

Having dreams, wanting to work or study, etc. helps them believe that they can have a better future and that their current situation is temporary:

*“I would like to have this house (a very luxurious house), bring up children …” HS*

*“I like to work with old people. My dream is to work with old people, take care of old people or to clean or take care of little children, you know” JF*

*“I want to learn more, study more, and know more new things. Before I didn’t know, now I want to learn more.” KS*

*“I’ve always liked business. But of course, my idea was to come here because you can earn much more than there and I said, so go there and I’ll start a business, which is what I have always wanted, to have a business too.” XB*

*“And now my dream is to find work, go with my children so they can see their grandmothers and I see my mother, my family, and then come back. That is my dream.” SM*

#### Feeling useful to others

They like not always being the ones who ask and receive; being able to give and receive makes them feel better:

*“… pretty soon I know a little more than the woman next to me, so I help her, but good, I feel good, I even went home and said to my husband that yes, I felt good, I liked it.”*

### Community assets

Many of the women do not have family nearby, or have only their immediate family of husband and children. Therefore, topics related to internal family or gendered perspectives are discussed with women friends and/or neighbours.

#### Children

The women pointed to their children as important motivators to improve their knowledge level; in many cases, children provide the inspiration or objective of their whole life, and also give them the opportunity to interact with other people in the school setting and to widen their social circle.

*“… I have three boys, and each one teaches me a little. One teaches me a little, the other teaches me a little … Yes, I have dreamt that my children should finish their studies.” BD*

*“One boy is in 5*^*th*^*now and he knows a lot. And the other is in 2*^*nd*^*and he knows a lot. He corrects me so much. He teaches me to speak Catalan. … and he says, this is how you have to learn to do things.” CM*

*“I know that I have to fight for the children and all of that.” SM*

#### Interpersonal relationships

The women discussed the importance of social support and of “significant others”, defined as people who have (or have had) a deep influence in their lives or with whom they are (or have been) emotionally involved [54]. They consider their family and other women friends and/or neighbours who constitute their social support to be fundamental to their ability to survive the situations of vulnerability they experience:

*“Sometimes you have blood relatives who don’t help and other family but not blood and they help you.” HS*

*“I know a woman who also is from Burkina who lives on Santa Eugenia Street. … She is very old but I admire her like a sister, not like my friend, like my sister.” SM*

#### Family setting

The importance of the family was observed, along with the difficulties of conciliating family and employment expressed by participants. At times, the women have to prioritize family over their work life in order to ensure the wellbeing of the family, especially the care of children so they can make progress:

*“My first girl, many times I think, yes I need to work but then if I go to work and what do I do, leave her to one side? I need to take her to the doctor and I can’t go with her. … But if I don’t work I also can’t do anything because she asks me for things I can’t give her.” XB*

*“But the boys, you can’t do this work, you can’t, they get out of school and that’s it, they will go get lost. I don’t want that.” BD*

### Institutional assets

Social and/or employment-related services provided important economic or other resources, such as public spaces, under the umbrella of institutional supports:

*“Since we ended up here, I will go to the Youth Office in –what is it called? – In Santa Eugenia Street, they help to make a résumé. There if you go they have computers like the university, and there is a girl to help you know how to get started.” JF“No. I have a computer but I don’t have internet. I go with the children to the library.” KS*

**Fig. 1:**
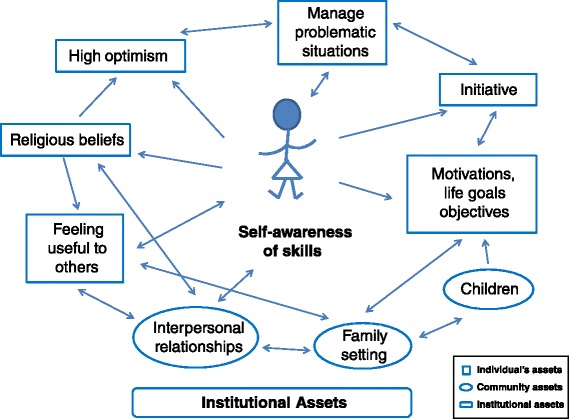
Relationships between assets classifications and self-awareness of skills. At a second level of analysis, self-awareness of skills was linked to a person’s description of characteristics or resources classified as individual or community assets, according to Morgan, Davies, and Ziglio [36]

At a second level of analysis (Fig. 1), we observed that self-awareness of skills was linked to a person’s description of herself as being optimistic, having religious beliefs, and having motivations and objectives in life, for herself, her family or her children. Being motivated helped the women to persist in doing or learning things that could be useful in confronting difficult situations. Another self-awareness skill was feeling useful to others, whether this was due to religious beliefs about their role in life or to the importance of the mutual support of interpersonal relationships.

## Discussion

Basing our analysis on the salutogenic approach [26] in a population of immigrant women, the results corroborate the SOC definition, both in general and in each of the three dimensions:

1. Comprehensibility allows a person to have a life that is structured, predictable and explainable. In contrast, the immigrant woman’s life is unpredictable and full of uncertainties, both economic and social, such as whether the immigrant will ever see her family or home country again.
2. Manageability, described as the resources that make it possible to have this life, consists of the resources participants possess: they know how to sew and/or cook great food, they are good managers of the household economy (maximizing every cent), and they know how to seek out external resources such as the public library and other institutional resources and support.
3. Meaningfulness, understood as challenges to be met and ways to remain motivated in life, could also be described in our population as challenges and dreams: learning, having a job, and making a home in the most decent conditions they can manage to provide for the family.

These results coincide with factors identified by other authors that favour or protect a woman’s wellbeing. These include describing what they know in terms of their life skills, not based only on their formal education, and knowing the importance of their role and responsibilities as mothers [40, 55].

Our main finding was that, despite the many challenges they face, the immigrant women from many cultures who participated in our study have many self-described assets: optimism, capacity for struggle and for initiative, religious beliefs, and social support. They have assumed an essential family role as caregivers for their children, always prioritizing family over the working world, and clearly considered their children the motivators or the objective of many of their dreams.

The optimism and perception of control described by study participants are aligned with the factors reported by Grote et al. as protectors against stress in a study of women with socioeconomic problems [56]. Our study reaffirms the social support of family and friends as protective factors against mental health problems and the importance of high-quality affective relationships (husband, friends and neighbours) previously reported by other authors [40, 55], and supports Cohen’s observation that not only the quantity but also the quality of this social support must be taken into consideration [57]. In addition, the role of mothers and caregivers was an important motivation that encouraged participants to carry on, not only for themselves but to fight for a better future for their children, which was one of their dreams – as in earlier studies [58].

At the same time, participants reported that their religious beliefs help them to accept and/or to confront the acculturative stress they experience as immigrants. This result coincides with a finding that Latin American immigrants in California report an inverse relationship between perceived stress and religious faith [59]. The assets model allows the creation of new strategies to promote health, based on knowledge of individual capabilities and community resources [35].

As Kretzmann and McKnight point out, however, a focus on the assets or GRR of vulnerable groups does not mean that a group has all the external resources they need [60]. The idea is not only to highlight the needs of these groups but also to recognize –and help them recognize in themselves– their own internal, external and community resources, in order to make better use of them and increase their self-esteem and SOC. Antonovsky discussed the need for holistic and integrated study of each individual, and for individual and community empowerment to reduce health inequities and provide a different –positive– view of immigrant women. However, we would emphasize that as of 2013 only 12 % of European countries had national policies designed to address these inequities [12]. According to Morgan and Ziglio, describing the assets available to immigrant women should enable a new approach to social and health policies oriented toward this group and serve as a tool to gain a better understanding of the causes of health inequities and the mechanisms by which they occur, in order to develop the new strategies required to remove them [61]. Some authors have described the need to reinforce policies, not only to cover the basic needs of vulnerable individuals and families, but also to empower them to meet their own needs [62]. One approach would be to reorient health promotion policies toward a salutogenic perspective, using asset models in the most vulnerable groups to empower the whole person by creating networks and synergies among all the different agents involved in supporting them. In addition, new policies designed to move toward equity in health resources require multi-sector interventions that encompass various determinants of health. These include reducing long-term unemployment, improving workplace conditions, reducing social segregation and increasing social participation in civic life, improving the physical environment and public transportation, ensuring equal access to healthcare services, and encouraging community-based capacity-building in health promotion. It is also important to include the affected communities, and specifically immigrant women, in the design and development of these policies from the outset, focusing on their needs but also on their assets.

The main limitation of the present study was the very basic level of Spanish language skills of many participants; if a participant had difficulty expressing her thoughts or feelings, this could have affected part of the study. This also complicated data analysis and interpretation because the participants tended to use very short sentences, which could have lost some meaning when extracted from the broader context. To address this limitation, multiple qualitative techniques were used, adapting them to the characteristics of the study participants in an effort to achieve the overall research objective. However, the linguistic aspect of the study was both a challenge and a strength because it was an opportunity to apply best practice research techniques used in populations with language-related difficulties. A major strength of the theoretical framework was that it allowed new constructs of “sources of health” to surface, rather than focussing on a diagnosis of the well-known challenges faced by immigrant populations. As these women were not strangers to each other, the first phase of the study seemed to help them encourage each other, particularly when language was a barrier. In particular, the photovoice session was very helpful in encouraging the women with the most limited language skills to express themselves. In future research with this population, photovoice methodology could be the first approach, and could be expanded somewhat. Once a rapport had been established with the researchers, the personal interviews were very useful in allowing each individual participant to have her own “last word” on the subjects that most interested her. A final strength of the study was the diversity within the group of immigrant women who participated. They were not only from different countries in North Africa, sub-Saharan Africa and Latin America but also differed in their cultural and educational backgrounds, marital status, and immigrant situation in Spain.

## Conclusions

From a salutogenic perspective, we can classify the main GRRs or assets of immigrant women as individual, community, and institutional. Individual resources included the ability to manage difficult situations, the capacity for initiative, the importance of religious belief, and high levels of optimism, personal motivation, and feeling useful to others. Community GRRs include the importance of children, family, and interpersonal relationships. In the institutional group, participants highlighted the support they had received.

The women who participated in this study provided evidence that immigrant women have assets and know how to use them, shedding light on a new asset-based approach to health promotion interventions to reduce health inequities, rather than focusing only on the needs of the groups involved. The results indicate the necessity of investing more effort and resources in social, educational, and health policies and gathering in-depth evidence of the effectiveness of interventions based on this new approach.

## Abbreviations

GRR, general resistance resources; SOC, sense of coherence
